# Harnessing Pandemonium: The Clinical Implications of Tumor Heterogeneity in Ovarian Cancer

**DOI:** 10.3389/fonc.2015.00149

**Published:** 2015-06-30

**Authors:** Sarah P. Blagden

**Affiliations:** ^1^Department of Oncology, Churchill Hospital, University of Oxford, Oxford, UK

**Keywords:** ovarian cancer, heterogeneity, chemotherapy resistance, tumor evolution, HGSOC, clonal evolution, diversity, ITH

## Abstract

Heterogeneity has emerged as a key feature of ovarian cancer between different ovarian cancer subtypes; within single ovarian cancer subtypes; and within individual patient tumors. At the genomic level, with the advent of ultra-deep sequencing technologies alongside RNA-Seq, epigenomics, and proteomics, the complexity surrounding heterogeneity has deepened. Here, we summarize the emerging understanding of heterogeneity in cancer as a whole and the key discoveries in this area relating to ovarian cancer. We explore the therapeutic limitations and possibilities posed by heterogeneity and how these will influence the future of ovarian cancer treatment and research.

## Background

Cancer cells within a tumor, rather than being identical, differ greatly from each other in their morphology, gene expression patterns, metabolism, rates of motility, proliferation, and/or metastatic potential (1). This is termed as intratumoral heterogeneity (ITH). As a malignant tumor is believed to arise from a single abnormal cell of origin, the mechanism by which this divergence occurs has been debated for over 40 years. The pathologist Peter Nowell proposed a “clonal evolution model” in 1976 whereby an initial neoplastic event in one or more cells confers them with a survival advantage. Other genetic mutations affecting subsequent generations of cells enhance their survival and ultimately lead to a highly aneuploid and aggressive mature tumor population (2). Nowell’s stochastic explanation was challenged following the discovery of bone marrow progenitor cells in the 1960s and 1970s [e.g., Ref. (3)] leading to the “stem cell model” whereby cancers comprise a hierarchy of tumorigenic stem cells continuously spawning non-tumorigenic progeny [reviewed by Ref. (4)]. Subsequent decades of research, advantaged by tools such as high throughput genomic, epigenetic, and proteomic sequencing, have revealed a much more complex picture. Cancers may arise from more than one founder cell, contain subpopulations driven by stochastic, stem cell, and mixed hierarchies, and are exposed to post-transcriptional and environmental influences (such as hypoxia and acidosis) resulting in their heterogeneity (5–7). Heterogeneity is therefore both genetic, in which mutations are branded into the genome and carried forward into subsequent generations, and responsive, whereby expression of genes is altered by environmental factors or in response to selective pressures. We can conclude that, by the time a tumor is “established” (even at 1 cm^3^ in size, it is estimated to contain one billion cancer cells), each mature cell carries founder and acquired somatic mutations and is highly heterogeneous.

In 1970s, Heppner and others proved the concept that heterogeneity could influence the chemotherapy response. Heppner used syngeneic xenografts bearing tumors derived from a single human breast cancer cell line to show that, despite sharing the same parent cell, the established xenograft tumors had differential sensitivity to the three main chemotherapy agents used for breast cancer: cyclophosphamide, methotrexate, and 5-fluorouracil (8). She noted that the primary tumor at the site of implantation within these xenografts was invariably more sensitive to chemotherapy than were the metastatic deposits. This divergence between primary and metastatic sites is termed as intertumoral heterogeneity.

In 1970s and 1980s, when cytotoxics were the main anti-cancer treatments available, the discovery of tumor heterogeneity was little more than a stumbling block to the development of predictive chemosensitivity assays. Cancer had long been believed to be a disease of the genome, confirmed by the association between H-Ras mutation and bladder cancer in 1982 (9). Identification of the entire spectrum of genes involved in cancer was impossible; sequencing even a single gene was costly and time-consuming and the total number of genes within the genome was unknown. This situation was transformed in a race between academia and industry to annotate the human genome. The academic International Human Genome Sequencing Consortium (IHGSC) used a bacterial artificial chromosome (BAC)-to-BAC sequencing technique in which chromosomes from anonymous donors were cut into pieces, inserted into BAC clones to generate a library before being mapped, and then sequenced. Their industrial competitor Celera (founded by the geneticist and entrepreneur Craig Venter) opted for a faster, shotgun-sequencing approach in which pieces of DNA were inserted into a plasmid library before sequencing. The academics were first to release a working draft of the genome sequence on the internet in 2000. This was followed in 2001 by publications from IHGSC and Celera (10, 11), respectively. IHGSC published a final, finished sequence in 2004 (12). Annotation of the human genome and that of other organisms heralded an “era of genomics” and enriched our understanding of human evolution and biology.

From the oncology perspective, defining the human genome sequence provided an essential reference against which the “cancer genome” could be compared. In 2004, a census of 291 gene mutations linked to cancer was published (13). The first intertumoral comparison used whole exome sequencing in tumors from 11 patients with colorectal and 11 patients with breast cancer (14). Initially, the volume of data generated in these types of comparative studies limited the number of tumors that could be sequenced. This was overcome with the arrival of DNA sequence enrichment technologies. Since then, the number of tumor types to have been genetically characterized by whole exome and (to a lesser extent) whole genome sequencing has expanded dramatically (http://www.sanger.ac.uk/genetics/CGP/cosmic/papers/). Collaborative initiatives, such as the International Cancer Genomics Consortium (ICGC), the Cancer Genome Atlas Project (TCGA), and the Cancer Genome Project (CGP), have pooled the sequencing data enabling more comprehensive genetic characterization of cancer types (15). This has expanded the list of known oncogenic drivers and gene mutations regulating pathological signaling pathways, information that can be used to better define, identify, or therapeutically target cancer [(13) and subsequently COSMIC database]. Although around 800,000 somatic mutations have been identified so far, the vast majority of these are non-physiologically significant (passenger mutations) with the more potent driver mutations being of low frequency or as yet undetected in many cancers. When DNA sequencing data were taken into context using copy number analysis, RNA expression (cDNA sequencing or RNA-Seq), and epigenetic profiling, the list of mutations functionally relevant to each cancer was reduced to an average of 30–60 (16).

The identification of gene mutations associated with cancer accelerated the drive to replace cytotoxics with “targeted therapies” (usually small molecule inhibitors or monocolonal antibodies). This yielded some notable success stories in which solid cancers with clear oncogenic drivers, such as the HER2 receptor in breast cancer and mutant B-Raf in melanoma, showed dramatic clinical responses to the targeted therapies Herceptin (trastuzumab) and PLX4032 (vemurafenib), respectively (17, 18). However, the majority of targeted agents were less successful, with a short-lived or absent clinical response (19). This was particularly true in epithelial ovarian cancer (EOC). Using the example of trastuzumab, even in EOC tumors in which HER2 was upregulated, a lack of response to the drug was observed [Teplinsky and Muggia (20)]. This implied that driver mutations or amplifications identified in one tumor were not necessarily conserved across all cancers. An overview of the first wave of targeted therapies is sobering: of 71 drugs approved by the FDA between 2002 and 2012, the median gain to patients’ overall survival was only 2.1 months (21). It was the realization that a therapeutic target identified on a single, archived tumor sample was perhaps not representative of the current molecular status of a cancer that reactivated the issue of tumor heterogeneity.

## Tracking Heterogeneity in Epithelial Ovarian Cancer – The Precursor Lesion

Epithelial ovarian cancer has long been considered a histologically heterogeneous cancer as it comprises at least five distinct histological subtypes, the most common and well-studied being high-grade serous ovarian cancer (HGSOC). At a molecular level, EOC is also heterogeneous (22). In contrast to cancers that present in anatomic positions where they are quickly detectable, EOC is most commonly diagnosed once it has spread outside the pelvis and into multiple sites around the abdominal cavity. It is probable that the significant lead-time between developing and detecting EOC provides the tumor an opportunity to undergo significant clonal expansion. This is supported by the stage-by-stage deterioration in prognosis from a 90% 5-year survival at stage I to only 4% if diagnosed at stage IV (cancerresearchuk.org). An important component to “tracking” the accumulation of genetic mutations that drive the formation and subsequent progression of a cancer is in identifying its precursor lesion. For ovarian cancer, this has been a longstanding source of contention [summarized by Ref. (23)]. All subtypes of EOC were originally believed to arise from dysplastic squamous epithelial cells covering the ovary or inclusion cysts formed from invaginations of the ovarian surface epithelium (24). Subsequent pathological and epidemiological studies suggest distinct tissues of origin for the main EOC histotypes. For example, the low-grade endometrioid and clear cell histotypes of EOC are believed to be derived from endometriotic tissue that has migrated along the fallopian tube onto the ovary (25) and mucinous EOC from “Walthard nests,” benign clusters of epithelial cells with morphological similarities to urothelial tissue present at tubal–mesothelial junctions (26). The strongest precursor association has been made between HGSOC cancers and fallopian tube premalignant lesions termed as serous tubal intraepithelial carcinomas (STICs) located at the tubal–peritoneal junctions. This discovery came from pathological analyses of specimens collected at the time of prophylactic salpingo-oophorectomy in women with an inherited predisposition to ovarian cancer (27). STIC lesions have also been identified in the fallopian tubes of 70% patients with sporadic ovarian and serous peritoneal cancer implying its association is not limited to BRCA carriers (28). However, that STICs are precursors for all cases of HGSOC is still a matter of controversy and debate (29, 30). Of note, *TP53* mutations have been identified in STIC lesions and not in inclusion cysts implying that they might be an early event in HGSOC tumorigenesis and supporting STICs as *bone fide* precursor lesions (31). The finding of a precursor lesion not only facilitates preventative strategies but also gives an important starting point from which to map heterogeneity of the disease.

## Heterogeneity in Ovarian Cancer

The presence of heterogeneity in EOC was first formally described in 1979 when ploidy was quantified using metaphase spreads from cells extracted from patients’ ascites before and after chemotherapy. Thymidine incorporation in *ex vivo* cells was used to assess chemotherapy sensitivity. The authors concluded that there were significant differences in chromosome distribution after chemotherapy (although overall levels of ploidy were unchanged) and different chemosensitivity between tumors cells obtained from distinct metastatic sites (32). Later, with the dawn of recombinant DNA technology in the 1990s, genetic studies became more sophisticated. By analyzing the restriction fragment length polymorphisms, Ehlen and Dubeau (33) identified significant chromosomal loss of heterozygosity in chromosomal segments 3p, 6q, and 11p in EOC tumor tissue while Lee et al. (34) demonstrated allelic loss of chromosomes 6q, 11, and 17. Later, some of these sites were found to harbor tumor suppressor genes, such as chromsome 17, which is the genomic location of *BRCA1* and *TP53* (35). At this point, polymerase chain reaction (PCR) and fragment sequencing enabled the identification of mutations within known cancer-associated genes. Using this technique mutations were identified in BRCA and P53 (36, 37). Invariably, these analyses were conducted on single biopsy specimens. In a study published in 2007, multiple specimens were microdissected from paraffin-embedded tumors obtained from 22 patient samples and compared using microsatellite and single nucleotide polymorphism (SNP) analysis. Although all tumors were HGSOC and thus morphologically similar, there was considerable genetic heterogeneity observed between patients and between samples collected from the same patient, manifested as chromosome deletions (particularly chromosomes 13 and 17), microsatellite instability, and SNP variation (38).

Understanding the genetic composition of HGSOC was greatly enhanced with data from TCGA Research Network. Here genomic, epigenomic, and transcriptomic expression data from 489 patients with HGSOC were pooled and analyzed for mRNA, microRNA, DNA copy number, and sites of DNA methylation. This was correlated with parallel whole exome sequencing, hybrid affinity capture methods, and cliniopathological information. The results confirmed a 96% prevalence of mutations in *TP53*, 22% prevalence of *BRCA1* or *2* mutations, and seven other gene mutations at lower frequency (in 2–6% cases). When compared to 11 other cancer types, ovarian cancer was notable for its low number of non-synonymous mutations (39). There were, however, high degrees of somatic copy number alterations, which were attributed to mutations and promoter methylations affecting DNA homologous repair genes (40). This was endorsed in studies using paired-end next generation sequencing and high-density SNP arrays revealing high copy number and frequent structural variations (41, 42). Homologous recombination (HR) deficiencies are present in approximately 50% of HGSOC. The predominant contribution is from somatic BRCA mutations, but as translational data from the ARIEL2 study of the PARP inhibitor rucaparib demonstrated, loss-of-function mutations, or homozygous deletion of other HR genes, such as RAD51C also contribute (43). The TCGA results provided important insights into the transcriptional landscape of HGSOC. Although only one sample was analyzed from each patient’s primary tumor (prior to chemotherapy), it provides an essential basis for comparison, just as the human genome had done a decade before.

Against the backdrop of heterogeneity research, other groups have attempted to subclassify ovarian cancer, exemplified by work from David Bowtell’s group in Melbourne. Using microarray gene expression profiling on tumor tissue collected from 285 patients at the time of debulking surgery, they identified six molecular subtypes of ovarian cancer and correlated their findings with clinical outcomes (44). Four molecular subtypes of HGSOC were identified: C1 (high stromal response), C2 (immunoreactive), C4 (low stromal response), and C5 (mesenchymal); the C1 subtype having the worst survival outcome (45). It is noteworthy that this method of subclassification identified immunoregulatory and stromal genes, rather than those involved in DNA repair. For example, the C1 group was characterized by desmoplasia and over-expression of myofibroblast markers, such as ACTA2. In addition, there were variations in T-cells and leukocyte infiltration (CD3 and CD45 positivity, respectively) between subtypes, with C2 and C4 subtypes having high intratumoral and stromal CD3 positive cells compared to low intratumoral but high stromal CD3 infiltration in C1 tumors. This method of molecular subtyping highlights the impact of tumor environment on EOC outcome and provides an avenue for personalizing treatment. However, an important caveat is the contribution of tumor heterogeneity and the likelihood that a single microdissected specimen of tumor may not be representative of the entire disease. This applies as much to T cells as to tumor cells. Recent ultra-deep T cell receptor sequencing in multiple samples collected from patients with renal cell carcinoma revealed significant heterogeneity in T cell clones between disease sites (46). Encouragingly, the same might not be said for tumor-infiltrating lymphocytes (TILs) in EOC. In a study of high throughput sequencing of the T-cell repertoire in tumor samples taken during primary debulking surgery, the authors discovered considerable homogeneity in TILs at different sites of disease. Their findings imply that, in detecting overall TIL activity, a single tumor biopsy is adequate (47).

Investigations into tumor heterogeneity have evolved from demonstrating its existence to understanding its patterns of emergence and its clinical implications. In the context of EOC, this was specifically addressed in 2014 when 27 archived tumor biopsy samples taken from three patients with advanced (IIIC/IV) primary EOC were analyzed using a variety of methods, including transcriptome and mate-pair sequencing and targeted gene sequencing (48). Samples were collected from primary and distant metastatic sites and compared to matched normal tissue samples. Not only was a high degree of genetic heterogeneity observed (focusing on approximately 400 oncogenes) within each cancer, it was clear from the presence and absence of gene mutations in primary and secondary sites that disseminated cells continued to evolve independent of the primary tumor, forming genetically distinct metastatic deposits, which were themselves distinct from other metastases.

That the evolutionary pattern of cancer can be tracked by spatial tumor sampling has been demonstrated in pancreatic cancer (49), clear cell renal cancer (50), and HGSOC (51). In the latter study, 31 samples of HGSOC were taken from six patients prior to starting chemotherapy. Exome sequencing, copy number analysis, target amplicon deep sequencing, and gene expression profiling revealed extensive intratumoral genomic diversity. Although mutations in well-characterized genes, such as *PIK3CA, CTNNB1*, and *PDGFR*, were observed, they were not present in all samples. There was no reproducible pattern of evolution between patients; mutations to *TP53* being the only consistent genetic feature observed. In the Gerlinger study (50), fresh frozen samples were collected from primary and metastatic sites from four patients with renal cell cancer. A phylogenetic tree was assembled around the gene mutations identified in each patient. This demonstrated that, rather than occurring sequentially and in a linear fashion, mutations occurred in a branch-like pattern suggesting that tumor cells in secondary sites had undergone additional mutations that conferred them with ongoing metastatic potential. This branched model, visually represented by the figure of a tree, describes the mutations present in early clonal progenitors as being “trunk” mutations (52). An example in HGSOC would be the *TP53* mutations present in STIC lesions that are still ubiquitously present in metastatic tumor. In contrast, the branches represent later somatic events that occur following the separation of subclones. In HGSOC, an example of branch mutations would be *PIK3CA, CTNNB1*, and *PDGFR* mutations that are present in some, but not all, metastatic sites.

In a recent paper by Schwarz et al. (53), the extent of ITH was quantified and correlated with clinical outcome. Copy number profiles from 135 metastatic sites were obtained from 17 patients with HGSOC at the time of diagnosis and following chemotherapy. Heterogeneity was quantified as the degree of clonal expansion between tumors (CE-high and CE-low) using a novel algorithm called minimum event distance for intratumor copy number comparisons (MEDICC). This computed minimum event distances between copy number profiles to construct an evolutionary tree for each patient based on at least three tumor samples. The results demonstrated significant intratumor genetic heterogeneity. The authors demonstrated that in eight of nine patients, the tumor displayed a branched (rather than linear) pattern of evolution, again suggesting that mutations are ongoing even at metastatic sites. As the majority had received neoadjuvant chemotherapy, the authors then quantified CE in samples taken before and after chemotherapy and showed that chemotherapy induced only a minimal increase in copy number events, especially when compared to the tumor’s inherent heterogeneity. Those with higher CE had poorer progression-free (10.1 months compared to 12.7 months) and overall survival time (23.5 months compared to 42.6 months) compared to those with low CE. Because samples had been collected from two patients at the time of progression, the authors tracked the percentage of cells expressing a mutation in *NF1* (compared to *TP53*) in one patient. They demonstrated increasingly enriched expression of mutant NF1 in the fallopian tube primary, pre-treatment tissue, and post-treatment samples, and it was also present at relapse in ascitic fluid. This indicates, albeit in a single patient, that Nowell’s clonal diversity model is correct, a resistant subclonal cellular population is present at diagnosis, and that CE occurs between diagnosis and relapse.

## Clinical Implications of Tumor Heterogeneity in Ovarian Cancer

The most obvious clinical implication of tumor heterogeneity is that a molecularly targeted therapy, while being effective at one tumor site, may not be as effective at all of them. This would certainly correlate with the disappointing clinical activity of targeted therapies in EOC, with the exception perhaps of PARP inhibitors. There is even a case for avoiding targeted therapies altogether in the setting of highly heterogeneous tumors. However, findings from Schwarz et al. (53) and others indicate that some HGSOC cases are less heterogeneous than others, implying that low CE patients might benefit from a more targeted approach. Perhaps, a measure of tumor heterogeneity is as important as tumor staging in the pre-treatment assessment of the ovarian cancer patient. However, current methods to measure heterogeneity require biopsies and sequencing of tumor tissue from multiple disease sites to derive a CE (or equivalent) score, which is impractical in the routine clinical setting. It is possible that circulating tumor cells may provide a less invasive means of quantifying overall ITH, an avenue that is currently being explored in HER2 expressing breast cancer (54). Deep sequencing of circulating tumor DNA is another promising technique that has already been used to detect EGFR mutations acquired during chemotherapy resistance (55). However, the circulating milieu may not accurately represent that of the tumor and its surrounding stroma, as was demonstrated by Emerson et al. (47).

An alternative approach would be to generate a consensus “evolutionary tree” against which patients could be mapped to predict their tumor heterogeneity. Although the numbers of EOC patients in existing evolutionary studies are too low to make these assumptions, the findings so far show that, for most, the “tree” of EOC has a short trunk and many branches, representing early clonal expansion and high genomic instability. It is clear that algorithms like MEDICC should be used to objectively correlate the evolutionary maps obtained from more patients with HGSOC to observe for similarities in branching patterns and identify mutations that drive branching events and share a common pathway or physiological function. It is possible also that the time to branching is clinically relevant and tumors with wider trunks and more clonal mutations will derive greater benefit from targeted therapies. Greater understanding of the evolutionary tree of EOC may also help us screen for the emergence of mutations that drive branching episodes, define which mutations are actionable (i.e., treatable) and which are unrepresentative of the entire disease.

A more ambitious aim would be to reverse heterogeneity with the aim of restoring EOC to a more linear and therefore more chemotherapy-sensitive evolutionary pattern. This would depend on identifying and inhibiting the key drivers of heterogeneity. To address this, a number of prospective biopsy studies have been launched with the aim of mapping and tracking the origin and dissemination of HGSOC over time. This includes the British Translational Research *Ovarian Cancer* Collaborative (BRITROC) study conducted by the *BriTROC*. As the answer may not lie in the genome itself, it will be important to look at transcription efficiency, epigenetic changes, immunological and stromal contributions, protein expression, and splice variations. For example, in lung cancer, it has been shown that faulty RNA editing caused by mutations to the APOBEC cytidine deaminase protein is an early driver of heterogeneity (56). It is possible that cytotoxic chemotherapy increases tumor heterogeneity by augmenting genomic instability, although results from Schwarz et al. (53) suggest that this is not a major contributor. The finding of TIL homogeneity across all EOC tumor sites, albeit in a small cohort of patients, suggests the therapeutic proteins unaffected by genetic diversity, might prove to be the best cancer targets.

## Conclusion and Future Directions

Ovarian cancer is a highly heterogeneous tumor, evidenced by the failure of most targeted therapies to make significant impact against the disease. The degree of heterogeneity adversely correlates with outcome. Emerging data already show a strong and immediate pattern of clonal evolution (branching) in ovarian cancer, between diagnosis and recurrence, corresponding with the clinical picture of a tumor that is initially responsive but soon resistant to chemotherapy. The identification of STIC lesions in the fallopian tube as HGSOC precursors and advances in deep gene sequencing techniques have opened the door to longitudinal mapping studies and the comparison of heterogeneity across thousands (rather than tens) of patients. While delineating tumor heterogeneity might enhance patient stratification and prognostication, a more important scientific goal lies in the identification of heterogeneity drivers, not only in HGSOC, but in other less common histotypes. The therapeutic inhibition of these drivers has the potential to harness the pandemonium of genetic heterogeneity and improve response to cancer treatments.

## Conflict of Interest Statement

The author declares that the research was conducted in the absence of any commercial or financial relationships that could be construed as a potential conflict of interest.
